# Effect of air pollution on heart rate variability and pulmonary function in schoolchildren: a cross-sectional study, Cubatão, Brazil, 2017

**DOI:** 10.1590/S2237-96222026v35e20250368.en

**Published:** 2026-06-17

**Authors:** Fernando Rocha Oliveira, Laércio da Silva Paiva, Roberta Ribeiro Batista Barbosa, Fabiana Rosa Neves Smiderle, Italla Maria Pinheiro Bezerra

**Affiliations:** 1 Escola Superior de Ciências da Santa Casa de Misericórdia de Vitória, Mestrado em Políticas Públicas e Desenvolvimento Local, Vitória, ES, Brazil Escola Superior de Ciências Santa Casa de Misericórdia de Vitória Vitória ES Brazil; 2 Centro Universitário Faculdade de Medicina do ABC, Laboratório de Epidemiologia e Análises de Dado, Santo André, SP, Brazil Centro Universitário Faculdade de Medicina do ABC Laboratório de Epidemiologia e Análises de Dado Santo André SP Brazil

**Keywords:** Respiratory Function Tests, Air Pollution, Students, Autonomic Nervous System, Cross-Sectional Studies, Pruebas de Función Respiratoria, Contaminación del Aire, Estudiantes, Sistema Nervioso Autónomo, Estudios Transversales

## Abstract

**Objective:**

To assess the effect of air pollution on heart rate variability and pulmonary function in schoolchildren.

**Methods:**

This was a cross-sectional study with a convenience sample of schoolchildren living in a highly polluted area in Cubatão, São Paulo. Resting heart rate variability data were collected through cardiac monitoring, based on SDNN (standard deviation of NN intervals), rMSSD (root mean square of successive differences between NN intervals), and HFnu (high-frequency component in normalized units, an indicator of autonomic parasympathetic activity). In order to assess pulmonary function, spirometry was performed based on FEV1 (forced expiratory volume in the first second) and FEF25%-75% (forced expiratory flow between 25% and 75% of forced vital capacity). Pollutant concentration levels were provided by the São Paulo State Environment Company. Multiple linear regression was used to investigate associations between pollutant levels and physiological parameters, using Stata software version 12.0.

**Results:**

Seventy-seven schoolchildren were assessed. Increased sulfur dioxide was associated with a reduction in HFnu: -0.68 (95% confidence interval - 95%CI -1.27; -0.11) in the daily average, -0.67 (95%CI -1.26; -0.07) over two days, and -1.29 (95%CI -2.21; -0.38) over three days. Ozone was also associated with a -0.32 (95%CI -0.62; -0.02) reduction in HFnu. Regarding pulmonary function, increased nitrogen dioxide was associated with a 0.23% reduction in FEV1 and a 0.30% reduction in FEF25%-75%.

**Conclusion:**

Schoolchildren exposed to air pollution showed reduced heart rate variability and pulmonary function.

## Resumen

**Objetivo:**

Evaluar el efecto de la contaminación del aire en la variabilidad de la frecuencia cardíaca y la función pulmonar en escolares.

**Métodos:**

Estudio transversal con una muestra de conveniencia de escolares residentes en una zona altamente contaminada de Cubatão, São Paulo. Se recopilaron datos de variabilidad de la frecuencia cardíaca en reposo mediante monitorización cardíaca, utilizando las variables SDNN (desviación estándar de los intervalos NN), rMSSD (raíz cuadrática media de las diferencias sucesivas entre intervalos NN) y HFnu (componente de alta frecuencia en unidades normalizadas, un indicador de la actividad parasimpática autonómica). Para la evaluación de la función pulmonar, se realizó espirometría con las variables FEV1 (volumen espiratorio forzado en el primer segundo) y FEF25%-75% (flujo espiratorio forzado entre el 25% y el 75% de la capacidad vital forzada). Las concentraciones de contaminantes fueron proporcionadas por la Compañía Ambiental del Estado de São Paulo. Se utilizó la regresión lineal múltiple para investigar las asociaciones entre los niveles de contaminantes y los parámetros fisiológicos, utilizando el software Stata versión 12.0.

**Resultados:**

Se evaluaron 77 escolares. El aumento del dióxido de azufre se asoció con una reducción de la HFnu: -0,68 (intervalo de confianza del 95% - IC95% -1,27; -0,11) en el promedio diario, -0,67 (IC95% -1,26; -0,07) en dos días y -1,29 (IC95% -2,21; -0,38) en tres días. El ozono también se asoció con una reducción de la HFnu de -0,32 (IC95% -0,62; -0,02). En cuanto a la función pulmonar, el aumento del dióxido de nitrógeno se asoció con una reducción del 0,23 % en el FEV1 y una reducción del 0,30 % en el FEF25%-75%.

**Conclusión:**

Los escolares expuestos a la contaminación atmosférica mostraron una menor variabilidad de la frecuencia cardíaca y una función pulmonar reducida.

## Introduction

Air pollution is a complex combination of different gaseous and particulate components that result in various effects on human health [1]. In 2016, 300 million children worldwide breathed toxic air that exceeded international guidelines by six times or more [2]. Children are more vulnerable to air pollution because, on average, they breathe faster than adults, absorb more air relative to their body weight [2] and have less developed lung metabolic capacity compared to adults, making them more susceptible to air pollutants [3].

In 2017, respiratory infections accounted for 8.8% of all deaths in children aged 5 to 14 years. Among these deaths, it is estimated that 36% are attributable to exposure to air pollution, based on the population attributable fraction [4]. In schoolchildren aged 7 to 13 years living in industrial areas, exposure to pollutants such as particulate matter with a diameter of less than 2.5 micrometers and nitrogen oxides from industries was associated with reduced lung function, and exposure to particulate matter pollutants with a diameter of less than 2.5 micrometers was also related to dry cough [1].

There is scientific evidence on exposure to particulate matter and cardiovascular morbidity and mortality in adults, as well as long-term effects on the cardiovascular health of children [5-6]. The effects of exposure to particulate matter and cardiovascular effects on the reduction of heart rate variability have been studied. For every 10 μg/m^3^ increase in particulate matter with a diameter of less than 2.5 micrometers, there were 2% reductions in heart rate variability measurements [5]. In this regard, no studies were found that related particulate matter air pollution to heart rate variability in children [5].

Most investigations into air pollution are related to road traffic, and it is often difficult to differentiate the effects of this from those resulting from industrial emissions [7]. Specifically in relation to health, studies mainly link air pollution to problems with lung function [8-9]. The effects of industrial air pollution on lung function, respiratory symptoms and heart rate variability in children are poorly investigated. Cubatão, located in the state of São Paulo, is one of the most important industrial centers in Brazil, and was considered, between the 1970s and 1980s, to be one of the most polluted cities in the world [10].

Heart rate variability is widely used in the literature as a non-invasive marker of physiological stress and reflects the modulation of the autonomic nervous system, while pulmonary function represents the organ system most directly affected by inhalation of air pollutants. Therefore, they provide sensitive and complementary parameters for assessing the effects of environmental exposure.

The objective of this study was to assess the effect of pollution on heart rate variability and pulmonary function in schoolchildren exposed to air pollution in Cubatão in 2017.

## Methods

### Design

This is a cross-sectional study conducted between August and October 2017, in Cubatão, in southeast Brazil. 

### Setting

Cubatão is located in the Baixada Santista Metropolitan Region, 75 km from the state capital São Paulo. It has an area of ​​142.3 km² with a tropical climate (hot and humid), and is considered a major industrial center in the state of São Paulo. It was selected due to its historical concentration of industries and the significant environmental challenges it has faced over the decades, making it a unique setting for investigating exposure to air pollution.

### Participants

The study included schoolchildren aged 8 to 11, both male and female, enrolled at public elementary schools in Cubatão and who had lived in the municipality for at least six months. 

Schoolchildren were excluded if they were classified as obese according to the Food and Nutrition Surveillance System classification with a body mass index Z-score greater than 2 [11], if they had any previously reported or diagnosed disease (asthma, previous allergies, tuberculosis), disabilities that prevented the assessment procedures from being performed, use of any medication (antihistamines, antipyretics, antibiotics) and heart rate variability recording errors greater than or equal to 5%.

### Variables

The anthropometric variables were weight, height and hip, waist and neck circumferences, and waist-to-hip ratio.

The following time domain indices were used to analyze heart rate variability: Mean RR (mean of all RR intervals), SDNN (mean standard deviation of normal-to-normal RR intervals), Mean HR (mean heart rate of the series), rMSSD (root mean square of successive differences between normal RR intervals) and frequency domain indices - LFnu (low-frequency normalized units) and HFnu (high-frequency normalized units) [12,13].

The following indices were used to analyze pulmonary function: forced vital capacity (FVC), forced expiratory volume in the first second (FEV_1_), Tiffeneau index (FEV_1_/FVC), and forced expiratory flow between 25% and 75% of forced vital capacity (FEF_25%-75%_) [14].

Environmental and pollutant data were collected for particulate matter ≤10 μm (PM_10_), nitrogen dioxide (NO_2_), nitric oxide (NO), sulfur dioxide (SO_2_) and ozone (O_3_), temperature, and humidity.

### Data sources and measurement

The characteristics of the schoolchildren were obtained using a form prepared by the researcher and sent by the school to their parents/guardians, along with the validated International Study of Asthma and Allergies in Childhood questionnaire used to check for asthma and allergies, which was completed by parents/guardians in accordance with the questionnaire protocol [15].

Anthropometric measurements were taken according to standardized protocols available in the literature [16,17]. Information on age and race/skin color was also collected. Resting heart rate variability data were collected, in a seated position, for 25 minutes, followed by pulmonary function data collected using spirometry. All data collection took place at the schools, in classrooms previously made available for the research by the respective school administrators. The procedure was conducted by two researchers, one responsible exclusively for anthropometric measurements and data collection, and the other for heart rate variability and pulmonary function assessment, in order to standardize the procedures.

The time between two consecutive heartbeats was collected using the RS800CX heart rate monitor (Polar Electro, Finland), a validated, portable device with a 1kHz sampling rate. For the analysis of the intervals between two consecutive heartbeats, only recordings with more than 95% of sinus beats without artifacts were included in the studies [18].

Digital filtering was performed using Polar Precision Performance SW software (version 4.01.029), followed by manual filtering based on visual inspection of consecutive heartbeats (RR intervals) to eliminate ectopic beats and artifacts. Subsequently, 1,000 consecutive heartbeats were used for heart rate variability analysis, performed using Kubios HRV analysis software version 2.1 [12,19].

Spirometry testing was performed according to the guidelines of the American Thoracic Society [13]. The Koko PFT spirometer (nSpire Health, Longmont, Colorado, United States) was used, with daily calibration. The references adopted for the estimated values ​​were Polgar & Promadha [14]. Spirometry tests were performed by a single researcher, using an incentive screen provided by the device’s own software, in addition to standardized verbal prompts.

The environmental data were obtained from the São Paulo State Environment Company air quality system. Cubatão has three fixed air quality monitoring stations, located in the following neighborhoods: Centro (Rua Salgado Filho, 121, Parque Fernando Jorge), Vila Parisi (Rua Prefeito Armando Cunha, 70, Vila Parisi) and Vale do Mogi (Avenida Engenheiro Plínio de Queiróz, sem número, Jardim São Marcos). The data used were from the Centro air quality monitoring station. If daily data collection on any of the pollutant or temperature variables was below 75% at the Centro station, data collection was done at the next nearest station, that is, the Vila Parisi station and, subsequently, at the Vale do Mogi station.

### Bias control

Data collection was standardized and performed in the morning, between 7 am and 12 pm, in order to control for physiological variations related to the circadian rhythm, especially in measurements of heart rate variability and pulmonary function.

Heart rate variability and spirometry measurements were conducted by a single, previously trained researcher, ensuring standardization of procedures and avoiding interobserver variations. Before the official spirometry, all children underwent prior training, receiving the same verbal instructions and visual encouragement provided by the Koko equipment itself. Anthropometric measurements were performed using calibrated equipment.

### Sample size

The sample for this study was determined by convenience, based on the availability of participating schools and receipt of authorizations from legal guardians. Two public schools in Cubatão, located in regions with different levels of proximity to the industrial zone, were selected in order to represent distinct profiles of exposure to air pollution.

Initially, 153 schoolchildren were recruited in Cubatão. Seventy-six of them were excluded: 40 were obese, 20 had errors greater than 5% in heart rate variability measurement, 2 did not undergo spirometry, 10 had diagnosed asthma, and 4 were taking medication during data collection. The final sample consisted of 77 schoolchildren eligible for analysis.

### Statistical methods

Heart rate variability and spirometry assessments were recorded with the date (day/month/year) and time of measurement, as recorded automatically by the equipment. Similarly, air pollutant concentrations were obtained using a spreadsheet with hourly averages (24 measurements per day), identifying the date and time. This temporal structure allowed for matching between the databases, assigning to each participant the environmental values ​​corresponding to the day of physiological data collection.

Descriptive analysis of the data was performed using measures of central tendency and absolute and relative frequencies. Assuming that physiological effects on health may lag behind individual exposure, indicators were created from same-day and previous-day moving averages of pollutants and meteorological factors; that is, a two-day moving average is the average of the levels on the current day and the previous day.

Simple linear regression was performed to assess the relationship between the dependent variable and each air pollutant, and was also considered for the creation of multiple models. Multi-pollutant models were created for analysis. Multiple linear regression was used to assess associations between pollutants and the dependent variables (heart rate variability indices and spirometry).

The age, sex and temperature variables were used as adjustments for the models with heart rate variability. The height, age, sex and temperature variables were used to adjust the models with spirometric indices. A 5% significance level was used. Statistical analysis was performed using Stata statistical software version 12.0.

## Results

The sample was predominantly female, whose mean age was 10.09 years. The majority self-identified as mixed-race, with no participants identifying as Asian or Indigenous.

Mean body weight was 36.27 kg, while the mean height Z-score was 0.24, and the mean body mass index Z-score was 0.19. Mean waist circumference was 64.02 cm, hip circumference 74.65 cm, and neck circumference 28.44 cm. Among the clinical parameters, the mean heart rate was 87.50 beats per minute (bpm), and the mean respiratory rate was 19.58 breaths per minute (bpm). Mean peripheral oxygen saturation was 97.6 (Table 1).

**Table 1:** Means, standard deviations and relative and absolute freuqencies of the schoolchildren’s clinical characterization. Cubatão, 2017 (n=77)

Variables	Total	Statistics
Sex (number of participants; %)	77	
Female		46; 59.7%
Male		31; 40.2%
Race/skin color (number of participants; %)	77	
White		21; 27.2%
Black		10; 12.9%
Mixed-race		46; 59.7%
Asian		0; 0.0%
Indigenous		0; 0.0%
Age group (years)	77	
Mean (standard deviation)		10.09 (0.87)
Minimum-maximum		9.00-11.00
Weight (kg)	77	
Mean (standard deviation)		36.27 (6.38)
Minimum-maximum		24.30-51.60
Z-score - height (cm)	77	
Mean (standard deviation)		0.24 (1.03)
Minimum-maximum		-2.20-2.62
Z-score - body mass index (kg/m²)	77	
Mean (standard deviation)		0.19 (0.86)
Minimum-maximum		-1.97- 2.00
Waist circumfernce (cm)	77	
Mean (standard deviation)		64.02 (6.53)
Minimum-maximum		52.00-84.20
Hip circumfernce (cm)	77	
Mean (standard deviation)		74.65 (6.20)
Minimum-maximum		61.30-88.00
Neck circumfernce (cm)	77	
Mean (standard deviation)		28.44 (1.70)
Minimum-maximum		25.00-33.00
Heart rate (bpm)	77	
Mean (standard deviation)		87.50 (13.57)
Minimum-maximum		65.00-114.00
Respiratory rate (bpm)	77	
Mean (standard deviation)		19.58 (3.44)
Minimum-maximum		12.00-29.00
Peripheral oxygen saturation (%)	77	
Mean (standard deviation)		97.6 (1.0)
Minimum-maximum		96.0-99.0

The descriptive values ​​of the heart rate variability indices and spirometric indices showed that the mean RR of all RR intervals was 620 ms, while the mean HR of the series was 97.26 beats per minute. The mean standard deviation of the RR intervals (SDNN) was 53.2 ms, and the root mean square of the successive differences between the RR intervals (rMSSD) was 33.4 ms.

In the frequency domain, low frequency (LF) showed a median value of 63.10, while for the high frequency (HF) it was 36.70.

The predicted percentage values ​​in relation to lung function were: forced vital capacity (FVC), 86%; forced expiratory volume in the first second (FEV₁), 85%; Tiffeneau index (FEV₁/FVC), 108%; and forced expiratory flow between 25% and 75% of forced vital capacity (FEF25%-75%), 90% (Table 2).

**Table 2:** Medians and 95% confidence intervals (95%CI) of heart rate variability indices and pulmonary function. Cubatão, 2017 (n=77)

Variables	Median	95%CI
Heart rate variability indices		
Mean RR (ms)^a^	620.00	601.60; 639.50
Mean HR (1-min)^b^	97.26	94.40; 100.30
SDNN (ms)^c^	53.20	49.90; 56.10
rMSSD (ms)^d^	33.40	29.90; 35.90
LFnu^e^	63.10	60.20; 67.30
HFnu^f^	36.70	32.30; 39.40
LF/HF^g^	1.99	0.30; 6.40
Predicted pulmonary function percentages		
FVC^h^	86.00	83.00; 89.60
FEV_1_^i^	85.00	82.00; 88.60
FEV_1_/FVC^j^	108.00	106.00; 109.60
FEF_25%-75%_^k^	90.00	82.30; 93.00

a Mean RR (ms): mean RR interval in milliseconds; ^b^Mean HR (1-min): mean heart rate in beats per minute; ^c^SDNN (ms): standard deviation of all normal-to-normal RR intervals in milliseconds; ^d^rMSSD (ms): root mean square of successive differences in milliseconds; ^e^LFnu: low-frequency normalized unit; ^f^HFnu: high-frequency normalized unit; ^g^Low-to-high frequency ratio; ^h^FVC: forced vital capacity; ^i^FEV_1_: forced expiratory volume in the first second; ^j^Tiffeneau index (FEV_1_/FVC). ^k^FEF_25%-75%_: forced expiratory flow between 25% and 75% of FVC.

The associations between heart rate variability indices and exposure to air pollutants were assessed and are presented in Table 3. Particulate matter (PM₁₀) showed significant negative association with SDNN in the two-day moving average (β -0.59; 95%CI -1.10; -0.07) and three-day moving average (β -0.94; 95%CI -1.72; -0.15), indicating a reduction in overall heart rate variability as levels of this pollutant increased.

Among the other parameters, ozone showed positive association with the mean standard deviation of the RR intervals (SDNN) in the daily average (β 0.25; 95%CI 0.02; 0.47), suggesting an effect opposite to that of PM₁₀. With regard to the low frequency component (LF), significant positive association with sulfur dioxide was observed in all analyzed daily average models (β 0.68; 95%CI 0.10; 1.27), two-day moving average models (β 0.67; 95%CI 0.07; 1.26) and three-day moving average models (β 1.29; 95%CI 0.38; 2.21). High frequency (HF) showed significant inverse correlation with the same pollutant (β -0.68; 95%CI -1.27; -0.11, in the daily average, and β -1.29; 95%CI -2.21; -0.38, in the three-day moving average).

Ozone also showed positive association with the LF/HF ratio (β 0.03; 95%CI 0.004; 0.06), as did sulfur dioxide (β 0.06; 95%CI 0.01; 0.12, in the daily average, and β 0.15; 95%CI 0.06; 0.25, in the three-day moving average). These results indicated a predominance of sympathetic modulation in response to exposure to pollutants, especially sulfur dioxide, and a reduction in overall variability associated with fine particulate matter (Table 3).

Associations were found between spirometric indices and air pollutant levels in the sample assessed (Table 4). There was significant negative association between nitrogen dioxide and forced expiratory volume in the first second (FEV₁) in the daily average (β -0.23; 95%CI -0.43; -0.03), indicating a reduction in pulmonary function associated with exposure to this pollutant. Nitrogen dioxide also showed inverse association with forced expiratory flow between 25% and 75% of forced vital capacity (FEF_25%-75%_) (β -0.30; 95%CI -0.58; -0.03).

**Table 3:** Beta coefficients (β) and 95% confidence intervals (95%CI) of the regression analysis between heart rate variability and air pollutant levels in schoolchildren. Cubatão, 2017 (n=77)

Models^b^	Daily average		2-day moving average		3-day moving average	
	β^g^	95%CI^h^	β^g^	95%CI^h^	β^g^	95%CI^h^
SDNN^i^						
PM_10_^c^ (µg/m^3^)	-0.17	-0.56; 0.20	-0.59^a^	-1.10; -0.07	-0.94^a^	-1.72; -0.15
NO_2_^d^ (µg/m^3^)	0.02	-0.12; 0.16	0.07	-0.14; 0.30	-0.01	-0.42; 0.39
O_3_^e^ (µg/m^3^)	0.25^a^	0.02; 0.47	0.30	-0.05; 0.65	0.32	-0.07; 0.72
SO_2_^f^ (µg/m^3^)	0.10	-0.78; 0.99	-0.34	-1.25; 0.56	-0.78	-2.06; 0.49
rMSSD^j^						
PM_10_^c^ (µg/m^3^)	-0.003	-0.31; 0.30	-0.24	-0.56; 0.06	-0.35	-0.75; 0.05
NO_2_^d^ (µg/m^3^)	0.03	-0.10; 0.16	0.04	-0.17; 0.25	-0.03	-0.29; 0.23
O_3_^e^ (µg/m^3^)	0.10	-0.08; 0.29	0.10	-0.14; 0.35	0.06	-0.19; 0.31
SO_2_^f^ (µg/m^3^)	0.003	-0.69; 0.70	-0.36	-1.07; 0.34	-0.82	-1.89; 0.23
LFnu^k^						
PM_10_^c^ (µg/m^3^)	0.07	-0.29; 0.43	-0.22	-0.63; 0.18	-0.30	-0.93; 0.32
NO_2_^d^ (µg/m^3^)	-0.18	-0.38; 0.01	-0.12	-0.39; 0.14	-0.13	-0.44; 0.17
O_3_^e^ (µg/m^3^)	0.14	-0.10; 0.39	0.27	-0.03; 0.59	0.32^a^	0.02; 0.63
SO_2_^f^ (µg/m^3^)	0.68^a^	0.10; 1.27	0.67^a^	0.07; 1.26	1.29^a^	0.38; 2.21
HFnu^l^						
PM_10_^c^ (µg/m^3^)	-0.07	-0.43; 0.29	0.22	-0.18; 0.63	0.30	-0.32; 0.92
NO_2_^d^ (µg/m^3^)	0.18	-0.01; 0.38	0.12	-0.14; 0.40	0.13	-0.17; 0.44
O_3_^e^ (µg/m^3^)	-0.14	-0.39; 0.10	-0.27	-0.59; 0.03	-0.3^2^a	-0.62; -0.02
SO_2_^f^ (µg/m^3^)	-0.68^a^	-1.27; -0.11	-0.67^a^	-1.26; -0.07	-1.29^a^	-2.21; -0.38
LF/HF^m^						
PM_10_^c^ (µg/m^3^)	0.005	-0.02; 0.03	-0.02	-0.05; 0.01	-0.02	-0.07; 0.03
NO_2_^d^ (µg/m^3^)	-0.009	-0.02; 0.003	0.002	-0.02; 0.02	-0.003	-0.03; 0.02
O_3_^e^ (µg/m^3^)	0.008	-0.02; 0.03	0.03	-0.005; 0.06	0.03^a^	0.004; 0.06
SO_2_^f^ (µg/m^3^)	0.06^a^	0.01; 0.12	0.08^a^	0.02; 0.14	0.15 ^a^	0.06; 0.25

a p-value<0.05; ^b^Model adjusted for mean temperature, age and sex; ^c^PM_10_: particulate matter ≤10 μm in micrograms per cubic meter; ^d^NO_2_: nitrogen dioxide in micrograms per cubic meter, ^e^O_3_: ozone in micrograms per cubic meter; ^f^SO_2_: sulfur dioxide in micrograms per cubic meter, ^g^regression β-coefficient estimated by the model; ^h^95%CI: 95% confidence interval for the β coefficient; ^i^SDNN: standard deviation of all normal-to-normal RR intervals in milliseconds; ^j^rMSSD: root mean square of successive differences between intervals in milliseconds; ^k^LFnu: low-frequency normalized unit; ^l^HFnu: high-frequency normalized unit; ^m^Low-to-high frequency ratio.

Ozone showed significant positive association with FEF_25%-75%_ in the two-day moving average (β 0.57; 95%CI 0.06; 1.08), suggesting a possible compensatory effect on small airway function. The particulate matter and sulfur dioxide variables did not show statistically significant association with the spirometric parameters assesed in the different exposure models, with coefficients ranging from β = -0.28 to 0.05 for particulate matter and from β = -0.14 to 0.87 for sulfur dioxide (Table 4).

**Table 4:** Beta coefficients (β) and 95% confidence intervals (95%CI) of the regression analysis between spirometric indices and air pollutant levels in schoolchildren. Cubatão, 2017 (n=77)

Models^b^	Daily average		2-day moving average		3-day moving average	
	β^g^	95%CI^h^	β^g^	95%CI^h^	β^g^	95%CI^h^
FVC^b^ predicted percentag^e^i						
PM_10_^c^ (µg/m^3^)	-0.28	-0.82; 0.24	-0.17	-0.57; 0.22	-0.15	-0.66; 0.35
NO_2_^d^ (µg/m^3^)	-0.17	-0.37; 0.02	-0.10	-0.33; 0.12	0.08	-0.23; 0.39
O_3_^e^ (µg/m^3^)	0.06	-0.28; 0.41	0.22	-0.12; 0.58	0.17	-0.15; 0.49
SO_2_^f^ (µg/m^3^)	0.27	-0.25; 0.80	0.58	-0.65; 1.83	0.87	-0.73; 2.48
FEV_1_^b^ predicted percenta^g^ej						
PM_10_^c^ (µg/m^3^)	-0.23	-0.75; 0.28	-0.08	-0.49; 0.34	-0.13	-0.75; 0.47
NO_2_^d^ (µg/m^3^)	-0.23 ^a^	-0.43; -0.03	-0.08	-0.34; 0.18	-0.06	-0.26; 0.40
O_3_^e^ (µg/m^3^)	0.14	-0.20; 0.49	0.36	-0.02; 0.74	0.27	-0.09; 0.64
SO_2_^f^ (µg/m^3^)	0.29	-0.20; 0.79	0.80	-0.36; 1.97	0.82	-0.77; 2.42
FEV_1_/FVC^b^ predicted percentag^e^k						
PM_10_^c^ (µg/m^3^)	0.07	-0.12; 0.26	0.15	-0.03; 0.33	0.02	-0.37; 0.41
NO_2_^d^ (µg/m^3^)	-0.09	-0.20; 0.02	0.009	-0.12; 0.14	-0.02	-0.18; 0.14
O_3_^e^ (µg/m^3^)	0.09	-0.07; 0.27	0.17	-0.03; 0.37	0.13	-0.08; 0.34
SO_2_^f^ (µg/m^3^)	0.009	-0.18; 0.20	0.27	-0.01; 0.57	-0.14	-0.72; 0.43
FEF_25%-75%_^b^ predicted percentag^e^l						
PM_10_^c^ (µg/m^3^)	-0.17	-0.78; 0.43	0.05	-0.58; 0.69	0.02	-0.72; 0.96
NO_2_^d^ (µg/m^3^)	-0.30 ^a^	-0.58; -0.03	-0.007	-0.38; 0.37	0.15	-0.30; 0.62
O_3_^e^ (µg/m^3^)	0.32	-0.08; 0.73	0.57 ^a^	0.06; 1.08	0.40	-0.12; 0.93
SO_2_^f^ (µg/m^3^)	0.07	-0.55; 0.70	0.86	-0.29; 2.02	0.70	-1.11; 2.52

a p-value<0,05; ^b^Model adjusted for temperature, age, sex and height; ^c^PM_10_: particulate matter ≤10 μm in micrograms per cubic meter; ^d^NO_2_: nitrogen dioxide in micrograms per cubic meter; ^e^O_3_: ozone in micrograms per cubic meter; ^f^SO_2_: sulfur dioxide in micrograms per cubic meter; ^g^β: regression coefficient estimated by the model; ^h^95%CI: 95% confidence interval for the β coefficient; ^i^FVC: forced vital capacity; ^j^FEV_1_: forced expiratory volume in the first second; ^k^FEV_1_/FVC: Tiffeneau index; ^l^FEF_25%-75%_: forced expiratory flow between 25% and 75% of the FVC.

## Discussion

This study assessed association between air pollutant levels and heart rate variability and pulmonary function in schoolchildren exposed to air pollution. The results demonstrated statistically significant associations between increasing pollutant concentrations and decreased heart rate variability and pulmonary function parameters.

In terms of variability, decreases were observed in the mean standard deviation of RR intervals (SDNN) with particulate matter ≤10 μm and ozone pollutants, low frequency (LF) with sulfur dioxide, high frequency (HF) with sulfur dioxide, and, finally, positive association in the ratio between the low-frequency and high-frequency components of heart rate variability (LF/HF) with ozone and sulfur dioxide. These findings suggest that exposure to pollutants is associated with heart rhythm alterations in children, resulting from autonomic nervous system dysfunction.

A study found that in 9 to 12 year-old children, short-term exposure to particulate matter was associated with reduced heart rate variability. This association was more pronounced in children with lower mitochondrial DNA content [20].

Reductions in the high frequency (HF) index are associated with parasympathetic withdrawal, while mean standard deviation of the RR intervals (SDNN) and low frequency (LF) indices are associated with sympathetic and parasympathetic nervous system activity [21]. However, it is not possible to distinguish whether changes in heart rate variability are due to increased sympathetic nervous system activity or parasympathetic withdrawal [17].

Understanding the behavior of the autonomic nervous system related to exposure to air pollution still presents divergence regarding sympathetic nervous system and parasympathetic nervous system decrease or increase [22-23]. These discordant findings probably result from differences in the characteristics of the studies regarding environmental exposure, study methods, populations addressed and pollutants analyzed [24].

Despite divergences about the behavior of the sympathetic and parasympathetic nervous systems, autonomic nervous system dysfunction is described as an important pathway in pathophysiological mechanisms. These explain the deleterious health effects caused by acute or chronic exposure to gaseous and particulate pollutants [25], associating them with changes in autonomic tone due to activation of pulmonary neural reflexes [26].

Most of these findings are related to studies in adults, the elderly, or a population at increased risk, such as heart patients and people with chronic diseases [27]. Studies relating heart rate variability in children are extremely scarce in the literature [20], which highlights the importance of our findings.

Our results for pulmonary function demonstrated significant association between nitrogen dioxide and decrease in forced expiratory volume in the first second (FEV_1_) and forced expiratory flow between 25% and 75% of forced vital capacity (FEF_25%-75%_), suggesting a deleterious effect on pulmonary function associated with exposure to air pollution.

Similar results have been described in previous studies [3,28-30] and from other places [10,28-30], further corroborating the results described in the literature. Most of the studies we analyzed associated the effects of air pollution with deleterious effects on children’s respiratory health and described particulate matter and nitrogen dioxide as the pollutants with the strongest association. In the case of sulfur dioxide, no consistencies with health effects were found. Ozone related to temperature appeared as a presumed protective factor for children’s respiratory health [30]. This presumed effect was observed in our results for FEF_25%-75%_ with the two-day moving average for ozone.

The health effects related to exposure to air pollution have been observed even when pollutants remain below the levels determined by legislation [29]. Decreased lung function was observed in children living in the city of Rio de Janeiro, even within values ​​considered acceptable by legislation for particulate matter ≤10 μm and nitrogen dioxide pollutants. This indicates that the acute effects of air pollution on children’s health may be present even below the air quality standard [29].

Children and adolescents are considered more vulnerable to deleterious effects caused by exposure to air pollution [1,7]. Lung and immune system immaturity factors, along with children’s higher ventilation rates and outdoor activities, make this group more susceptible to the effects of outdoor air pollution [3]. In general, the adverse effects on lung function in children are attributed to reduced alveolar formation and inflammation that leads to airway remodeling caused by exposure to air pollution [28].

Lung function in childhood is considered to be a strong predictor of lung function in adulthood, as development of lung function is observed until the end of adolescence in females, while in males it can take until they are approximately 20 years old, although in a smaller proportion than in childhood [3].

In the United States, in 2015, a significant improvement in lung function was observed in the 11-15 year age group due to a decrease in atmospheric pollutants. It was suggested that the improvement in lung function growth observed in children less exposed to pollutants may persist into adulthood, reducing the risk of respiratory problems [3].

Despite the importance of the results we found, some limitations should be addressed, such as failure to verify certain confounding factors like passive smoking, the level of physical activity of the students studied, and the time they spent outdoors. Another limitation was not checking the days of the week on which the samples were collected, as this could interfere with the concentration and interaction of pollutants with heart rate variability and pulmonary function.

Further studies are needed in order to gain understanding of the relationship between the effects of air pollution exposure and heart rate variability and lung function in schoolchildren.

## Data Availability

The final and consolidated database used in this research will be available upon request via email to the corresponding author, subject to approval.
