# Supplementary material for: Effect of air pollution on heart rate variability and pulmonary function in schoolchildren: a cross-sectional study, Cubatão, Brazil, 2017
Source: Epidemiol Serv Saude. 2026 Jun 17;35:e20250368. doi: 10.1590/S2237-96222026v35e20250368.en (PMC13686411; doi:10.1590/S2237-96222026v35e20250368.en)
Supplement: Translation

# Translation: Efeito da poluição atmosférica na variabilidade da frequência cardíaca e da função pulmonar em escolares: estudo transversal, Cubatão, 2017

**DOI:** 10.1590/S2237-96222026v35e20250368.pt

**Authors:** Fernando Rocha Oliveira, Laércio da Silva Paiva, Roberta Ribeiro Batista Barbosa, Fabiana Rosa Neves Smiderle, Italla Maria Pinheiro Bezerra

**Affiliations:** 1 Escola Superior de Ciências da Santa Casa de Misericórdia de Vitória, Mestrado em Políticas Públicas e Desenvolvimento Local, Vitória, ES, Brasil; 2 Centro Universitário Faculdade de Medicina do ABC, Laboratório de Epidemiologia e Análises de Dado, Santo André, SP, Brasil

**Keywords:** Testes de Função Respiratória, Poluição do Ar, Estudantes, Sistema Nervoso Autônomo, Estudos Transversais

## Abstract

**Objetivo:**

Avaliar o efeito da poluição na variabilidade da frequência cardíaca e da função pulmonar em escolares.

**Método:**

Estudo transversal com amostra por conveniência de escolares residentes em área de alta poluição em Cubatão, São Paulo. Foram coletados dados de variabilidade da frequência cardíaca em repouso por monitoramento cardíaco, utilizando as variáveis SDNN (desvio-padrão dos intervalos NN), rMSSD (raiz quadrada da média dos quadrados das diferenças sucessivas entre intervalos NN) e HFnu (componente de alta frequência em unidades normalizadas, indicador da atividade parassimpática autonômica). Para avaliação da função pulmonar, foi realizada espirometria com as variáveis VEF1 (volume expiratório forçado no primeiro segundo) e FEF25%-75% (fluxo expiratório forçado entre 25% e 75% da capacidade vital forçada). As concentrações de poluentes foram fornecidas pela Companhia Ambiental do Estado de São Paulo. Utilizou-se regressão linear múltipla para investigar associações entre os níveis de poluentes e os parâmetros fisiológicos, pelo software Stata versão 12.0.

**Resultados:**

Foram avaliados 77 escolares. O aumento de dióxido de enxofre esteve associado à redução de HFnu: -0,68 (intervalo de confiança de 95% - IC95% -1,27; -0,11) na média diária, -0,67 (IC95% -1,26; -0,07) em dois dias e -1,29 (IC95% -2,21; -0,38) em três dias. O ozônio também se associou à redução de HFnu em -0,32 (IC95% -0,62; -0,02). Quanto à função pulmonar, o aumento de dióxido de nitrogênio foi associado à redução de 0,23% no VEF1 e de 0,30% no FEF25%-75%.

**Conclusão:**

Os escolares expostos à poluição atmosférica apresentaram redução da variabilidade da frequência cardíaca e da função pulmonar.

## Introdução

A poluição do ar é uma combinação complexa de diferentes componentes gasosos e particulados que resultam em vários efeitos à saúde humana [1]. Em 2016, 300 milhões de crianças em todo o mundo respiraram ar tóxico que excediam as diretrizes internacionais em seis vezes ou mais [2]. As crianças são mais vulneráveis à poluição do ar, pois respiram, em média, mais rápido que os adultos, absorvem mais ar em relação ao seu peso corporal [2] e tem menor desenvolvimento da capacidade metabólica pulmonar em comparação aos adultos, o que as tornam mais suscetíveis aos poluentes do ar [3].

Em 2017, as infecções respiratórias representaram 8,8% do total de mortes em crianças de 5 a 14 anos. Entre esses óbitos, estima-se que 36% sejam atribuíveis à exposição à poluição do ar, com base na fração atribuível populacional [4]. Em crianças em idade escolar de 7 a 13 anos residentes em áreas industriais, a exposição aos poluentes como material particulado com diâmetro inferior a 2,5 micrômetros e óxidos de nitrogênio de indústrias associou-se à redução da função pulmonar, enquanto o poluente material particulado com diâmetro inferior a 2,5 micrômetros também foi relacionado à tosse seca [1].

Há evidência científica sobre a exposição ao material particulado e a morbimortalidade cardiovascular em adultos, bem como os efeitos de longo prazo na saúde cardiovascular de crianças [5-6]. Foram verificados os efeitos da exposição ao material particulado e os efeitos cardiovasculares sobre a baixa da variabilidade da frequência cardíaca. A cada aumento de 10 μg/m^3^ no material particulado com diâmetro inferior a 2,5 micrômetros, ocorreram reduções de 2% nas medidas de variabilidade da frequência cardíaca [5]. Nesse contexto, não houve estudos que relacionaram poluição atmosférica por material particulado e variabilidade da frequência cardíaca em crianças [5]. 

A maioria das investigações sobre poluição do ar está relacionada ao tráfego rodoviário, sendo frequentemente difícil diferenciar seus efeitos daqueles decorrentes das emissões industriais [7]. Na saúde, os estudos relacionam a poluição do ar principalmente a problemas da função pulmonar [8-9]. Os efeitos da poluição do ar da indústria sobre a função pulmonar, os sintomas respiratórios e a variabilidade cardíaca em crianças são poucos investigados. Cubatão, localizada no estado de São Paulo, é um dos polos industriais mais importantes no país, sendo considerada, entre as décadas de 1970 e 1980, uma das cidades mais poluídas do mundo [10]. 

A variabilidade da frequência cardíaca é amplamente utilizada na literatura como marcador não invasivo de estresse fisiológico e reflete a modulação do sistema nervoso autônomo, enquanto a função pulmonar representa o sistema orgânico mais diretamente afetado pela inalação de poluentes atmosféricos. Portanto, fornecem parâmetros sensíveis e complementares para avaliar os efeitos da exposição ambiental. 

O objetivo deste estudo foi avaliar o efeito da poluição na variabilidade da frequência cardíaca e da função pulmonar em escolares expostos à poluição do ar em Cubatão em 2017. 

## Métodos

### Delineamento

Trata-se de estudo transversal realizado entre agosto e outubro de 2017, em Cubatão, sudeste do Brasil. 

### Contexto

Cubatão está localizada na Região Metropolitana da Baixada Santista a 75 km da capital São Paulo. Possui território de 142,3 km² com clima tropical (quente e úmido), sendo considerada grande polo industrial do estado de São Paulo. Sua escolha se deve à sua histórica concentração industrial e aos desafios ambientais significativos que enfrentou ao longo das décadas, sendo cenário único para investigar a exposição à poluição atmosférica.

### Participantes

Foram incluídos escolares de 8 a 11 anos, dos sexos masculino e feminino, matriculados nas escolas públicas do ensino fundamental de Cubatão e que residissem no município há pelo menos seis meses. 

Foram excluídos os escolares classificados como obesos segundo a classificação do Sistema de Vigilância Alimentar e Nutricional com escore Z do índice de massa corporal maior que 2 [11], portador de alguma doença prévia declarada ou verificada (asma, alergias prévias, tuberculose), incapacidades que impedissem a realização dos procedimentos de avaliação, uso de algum medicamento (antialérgicos, antitérmicos, antibiótico) e erros de registros da variabilidade da frequência cardíaca maior ou igual a 5%.

### Variáveis

As variáveis antropométricas foram peso, altura, circunferências do quadril, da cintura e do pescoço e relação cintura-quadril.

Para análise da variabilidade da frequência cardíaca, foram utilizados os índices do domínio do tempo: Mean RR (média de todos os intervalos RR), SDNN (desvio-padrão médio dos intervalos RR), Mean HR (frequência cardíaca média da série), rMSSD (raiz quadrada das diferenças quadradas médias entre RR adjacente normal intervalos) e domínio da frequência - LFnu (baixa frequência em unidades normalizadas) e HFnu (alta frequência em unidades normalizadas) [12,13].

Para análise da função pulmonar, foram utilizados os índices capacidade vital forçada (CVF), volume forçado no primeiro segundo (VEF_1_), índice de Tiffeneau (VEF_1_/CVF) e fluxo expiratório forçado entre 25% e 75% da capacidade vital forçada (FEF_25%-75%_) [14].

Os dados ambientais e dos poluentes foram coletados do material particulado ≤10 μm (MP_10_), dióxido de nitrogênio (NO_2_), óxido nítrico (NO), dióxido de enxofre (SO_2_) e ozônio (O_3_), temperatura e umidade.

### Fonte de dados e mensuração

As características dos escolares foram obtidas por ficha elaborada pelo pesquisador e encaminhada pela escola aos responsáveis, juntamente com o questionário validado International Study of Asthma and Allergies in Childhood utilizado para verificação de asma e alergias que foi preenchido pelos responsáveis, de acordo com o protocolo do questionário [15]. 

Foram realizadas medidas antropométricas, conforme protocolos padronizados da literatura [16,17], e coletadas informações sobre idade, raça/cor da pele. Os dados da variabilidade da frequência cardíaca foram coletados em repouso, na posição de sedestação, durante 25 minutos e, em seguida, coletados os da função pulmonar, por meio da espirometria. Todas as coletas foram realizadas nas próprias escolas, em salas de aula previamente cedidas para a pesquisa pelas respectivas direções. O procedimento foi conduzido por dois pesquisadores, sendo um responsável exclusivamente pelas medidas antropométricas e coleta das fichas e o outro pela variabilidade da frequência cardíaca e pela avaliação da função pulmonar, de modo a padronizar os procedimentos.

A coleta dos tempos entre dois batimentos cardíacos consecutivos foi realizada pelo monitor de frequência cardíaca cardiofrequencímetro RS800CX (Polar Electro, Finlândia), um equipamento validado e de uso portátil com taxa de amostragem de 1kHz. Para análise dos intervalos entre dois batimentos cardíacos consecutivos, somente registros com mais de 95% de batimentos sinusais sem artefatos foram incluídos nos estudos [18]. 

A filtragem digital foi realizada pelo software Polar Precision Performance SW (versão 4.01.029), seguido da filtragem manual baseada em inspeção visual dos batimentos cardíacos consecutivo (intervalos RR) para eliminar batimentos ectópicos e artefatos. Em seguida, foram utilizados 1 mil batimentos cardíacos consecutivos para análise da variabilidade da frequência cardíaca, realizado pelo software Kubios HRV analysis versão 2.1 [12,19]. 

O teste de espirometria foi realizado segundo as diretrizes da American Thoracic Society [13]. Foi utilizado o espirômetro Koko PFT (nSpire Health*,* Longmont, Colorado, Estados Unidos), com calibração diária. As referências adotadas para os valores estimados foram Polgar e Promadha [14]. Os testes espirométricos foram realizadas por um único pesquisador, sendo utilizado um auxílio da tela de incentivo proveniente do próprio software do aparelho, além do comando verbal padronizado. 

Os dados ambientais foram obtidos do sistema de qualidade do ar da Companhia Ambiental do Estado de São Paulo. Cubatão possui três estações fixas de monitoramento localizadas nos bairros Centro (Rua Salgado Filho, 121, Parque Fernando Jorge), Vila Parisi (Rua Prefeito Armando Cunha, 70, Vila Parisi) e Vale do Mogi (Avenida Engenheiro Plínio de Queiróz, sem número, Jardim São Marcos). Os dados utilizados foram provenientes da estação de monitorização da qualidade do ar do Centro. Caso a recolha diária de dados sobre qualquer uma das variáveis ​​de poluentes ou temperatura fosse inferior a 75% na estação do Centro, a recolha de dados era efetuada na estação mais próxima a seguir, ou seja, na estação de Vila Parisi e, posteriormente, na estação de Vale do Mogi. 

### Controle de viés

Para minimizar potenciais fontes de viés, foram adotadas estratégias metodológicas ao longo do estudo. A coleta de dados foi padronizada e realizada no período da manhã, entre 7h e 12h, com o objetivo de controlar variações fisiológicas relacionadas ao ritmo circadiano, especialmente nas medidas de variabilidade da frequência cardíaca e função pulmonar. 

As coletas de variabilidade da frequência cardíaca e espirometria foram conduzidas por um único pesquisador previamente treinado, assegurando padronização dos procedimentos e evitando variações interobservador. Antes da realização da espirometria oficial, todas as crianças passaram por treinamento prévio, recebendo as mesmas instruções verbais e incentivo visual fornecidos pelo próprio equipamento Koko. As medidas antropométricas foram realizadas com equipamentos calibrados. 

### Tamanho da amostra

A amostra deste estudo foi definida por conveniência, conforme a disponibilidade das escolas participantes e o retorno das autorizações dos responsáveis legais. Foram selecionadas duas escolas públicas de Cubatão, localizadas em regiões com diferentes níveis de proximidade da zona industrial, com o objetivo de representar distintos perfis de exposição à poluição atmosférica.

Inicialmente, foram convocados 153 escolares. Foram excluídos 76 escolares em Cubatão, sendo 40 com obesidade, 20 com erros maiores que 5% da captação da variabilidade da frequência cardíaca, 2 escolares que não realizaram espirometria, 10 escolares com asma declarada e 4 em uso de medicação durante a coleta. A amostra final foi composta por 77 escolares elegíveis para as análises. 

### Métodos estatísticos

As avaliações de variabilidade da frequência cardíaca e espirometria foram registradas com data (dia/mês/ano) e horário da mensuração, conforme registro automático dos equipamentos. Da mesma forma, as concentrações dos poluentes atmosféricos foram obtidas por meio de planilha com as médias horárias (24 medições por dia), com identificação de data e hora. Essa estrutura temporal permitiu o pareamento entre os bancos de dados, atribuindo a cada participante os valores ambientais correspondentes ao dia da coleta fisiológica.

Para a análise dos dados, realizou-se análise descritiva, utilizando medidas de tendência central e frequências absoluta e relativa. Assumindo que os efeitos fisiológicos sobre a saúde podem apresentar defasagem em relação à exposição do indivíduo, criaram-se indicadores a partir das médias móveis do mesmo dia e dias anteriores dos poluentes e fatores meteorológicos, ou seja, uma média móvel de dois dias é a média dos níveis no dia concorrente e do dia anterior. 

A regressão linear simples foi realizada para avaliar a relação variável dependente com cada poluente do ar, além de ser considerada para criação dos modelos múltiplos. Modelos de multipoluentes foram criados para análise. Foi utilizado regressão linear múltipla para avaliar as associações dos poluentes sobre as variáveis dependentes (índices da variabilidade da frequência cardíaca e espirometria). 

As variáveis idade, sexo e temperatura foram utilizadas como ajustes para os modelos com a variabilidade da frequência cardíaca. As variáveis altura, idade, sexo e temperatura foram utilizadas para os modelos com os índices espirométricos. O nível de significância adotado foi de 5%. A análise estatística foi realizada com o software estatístico Stata versão 12.0.

## Resultados

A amostra foi composta predominantemente do sexo feminino, com média de idade de 10,09 anos. A maioria se autodeclarou parda, não havendo participantes de raça/cor da pele amarela ou indígena.

A média de peso corporal foi de 36,27 kg, enquanto o escore Z de altura apresentou média de 0,24, e o escore Z de índice de massa corporal de 0,19. Observou-se média de 64,02 cm para a circunferência da cintura, 74,65 cm para a circunferência do quadril e 28,44 cm para a circunferência do pescoço. Entre os parâmetros clínicos, a frequência cardíaca média foi de 87,50 batimentos por minuto, e a frequência respiratória média de 19,58 incursões por minuto. A saturação periférica de oxigênio apresentou média de 97,6 (Tabela 1).

**Tabela 1:** Média, desvio-padrão e frequências absoluta e relativa da caracterização clínica dos escolares. Cubatão, 2017 (n=77)

Variáveis	Total	Estatísticas
Sexo (nº de participantes; %)	77	
Feminino		46; 59,7%
Masculino		31; 40,2%
Raça/cor da pele (nº de participantes; %)	77	
Branco		21; 27,2%
Preta		10; 12,9%
Parda		46; 59,7%
Amarela		0; 0,0%
Indígena		0; 0,0%
Faixa etária (anos)	77	
Média (desvio-padrão)		10,09 (0,87)
Mínimo-máximo		9,00-11,00
Peso (kg)	77	
Média (desvio-padrão)		36,27 (6,38)
Mínimo-máximo		24,30-51,60
Escore Z - altura (cm)	77	
Média (desvio-padrão)		0,24 (1,03)
Mínimo-máximo		-2,20-2,62
Escore Z - índice de massa corporal (kg/m²)	77	
Média (desvio-padrão)		0,19 (0,86)
Mínimo-máximo		-1,97- 2,00
Circunferência da cintura (cm)	77	
Média (desvio-padrão)		64,02 (6,53)
Mínimo-máximo		52,00-84,20
Circunferência do quadril (cm)	77	
Média (desvio-padrão)		74,65 (6,20)
Mínimo-máximo		61,30-88,00
Circunferência do pescoço (cm)	77	
Média (desvio-padrão)		28,44 (1,70)
Mínimo-máximo		25,00-33,00
Frequência cardíaca (rpm)	77	
Média (desvio-padrão)		87,50 (13,57)
Mínimo-máximo		65,00-114,00
Frequência respiratória (rpm)	77	
Média (desvio-padrão)		19,58 (3,44)
Mínimo-máximo		12,00-29,00
Saturação periférica oxigênio (%)	77	
Média (desvio-padrão)		97,6 (1,0)
Mínimo-máximo		96,0-99,0

Nos valores descritivos dos índices da variabilidade da frequência cardíaca e dos índices espirométricos, a média de todos os intervalos RR (Mean RR) foi de 620 ms, enquanto a frequência cardíaca média da série (Mean HR) foi de 97,26 batimentos por minuto. O desvio-padrão médio dos intervalos RR (SDNN) apresentou mediana de 53,2 ms, e a raiz quadrada da média das diferenças quadradas entre intervalos RR adjacentes (rMSSD) foi de 33,4 ms.

No domínio da frequência, a baixa frequência (LF) apresentou valor mediano de 63,1, e a alta frequência (HF) de 36,70.

Os valores percentuais do previsto foram em relação à função pulmonar: capacidade vital forçada (CVF) de 86%, volume expiratório forçado no primeiro segundo (VEF₁) de 85%, índice de Tiffeneau (VEF₁/CVF) de 108% e fluxo expiratório forçado entre 25% e 75% da capacidade vital forçada (FEF_25%-75%_) de 90% (Tabela 2).

**Tabela 2:** Mediana e intervalo de confiança de 95% (IC95%) dos índices de variabilidade da frequência cardíaca e função pulmonar. Cubatão, 2017 (n=77)

Variáveis	Mediana	IC95%
Índices de variabilidade da frequência cardíaca		
Mean RR (ms)^a^	620,00	601,60; 639,50
Mean HR (1/min)^b^	97,26	94,40; 100,30
SDNN (ms)^c^	53,20	49,90; 56,10
rMSSD (ms)^d^	33,40	29,90; 35,90
LFnu^e^	63,10	60,20; 67,30
HFnu^f^	36,70	32,30; 39,40
LF/HF^g^	1,99	0,30; 6,40
Valores da porcentagem do previsto da função pulmonar		
CVF^h^	86,00	83,00; 89,60
VEF_1_^i^	85,00	82,00; 88,60
VEF_1_/CVF^j^	108,00	106,00; 109,60
FEF_25%-75%_^k^	90,00	82,30; 93,00

a Mean RR (ms): média dos intervalos RR em milissegundos; ^b^Mean HR (1/min): média da frequência cardíaca em batimentos por minuto; ^c^SDNN (ms): desvio-padrão de todos os intervalos RR normais em milissegundos; ^d^rMSSD (ms): raiz quadrada da média do quadrado das diferenças entre intervalos em milissegundos; ^e^LFnu: baixa frequência em unidades normalizadas; ^f^HFnu: alta frequência em unidades normalizada; ^g^Relação LF/HF; ^h^CVF: capacidade vital forçada; ^i^VEF_1_: volume expiratório forçado no primeiro segundo; ^j^Índice de Tiffeneau (VEF_1_/CVF). ^k^FEF_25%-75%_: fluxo expiratório forçado entre 25% e 75% da CVF.

As associações entre os índices da variabilidade da frequência cardíaca e a exposição aos poluentes atmosféricos foram avaliadas e apresentadas na Tabela 3. O material particulado (MP₁₀) apresentou associação negativa significativa com o SDNN na média móvel de dois dias (β -0,59; IC95% -1,10; -0,07) e de três dias (β -0,94; IC95% -1,72; -0,15), o que indicou redução da variabilidade global da frequência cardíaca com o aumento desse poluente.

Entre os demais parâmetros, o ozônio mostrou associação positiva com o desvio-padrão médio dos intervalos RR (SDNN) na média diária (β 0,25; IC95% 0,02; 0,47), o que sugeriu efeito oposto ao do MP₁₀. Para o componente baixa frequência (LF), observou-se associação positiva significativa com o dióxido de enxofre em todos os modelos analisados de média diária (β 0,68; IC95% 0,10; 1,27), média móvel de dois dias (β 0,67; IC95% 0,07; 1,26) e três dias (β 1,29; IC95% 0,38; 2,21). A alta frequência (HF) apresentou correlação inversa significativa com esse mesmo poluente (β -0,68; IC95% -1,27; -0,11, na média diária, e β -1,29; IC95% -2,21; -0,38, na média móvel de três dias).

O ozônio também mostrou associação positiva com a razão LF/HF (β 0,03; IC95% 0,004; 0,06), assim como o dióxido de enxofre (β 0,06; IC95% 0,01; 0,12, na média diária, e β 0,15; IC95% 0,06; 0,25, na média móvel de três dias). Esses resultados indicaram predomínio da modulação simpática diante da exposição a poluentes, especialmente ao dióxido de enxofre, e redução da variabilidade global associada ao material particulado fino (Tabela 3).

Foram apresentadas associações entre os índices espirométricos e os níveis de poluentes atmosféricos na amostra avaliada (Tabela 4). Houve associação negativa significativa entre o dióxido de nitrogênio e o volume expiratório forçado no primeiro segundo (VEF₁) na média diária (β -0,23; IC95% -0,43; -0,03), indicando redução da função pulmonar associada à exposição a esse poluente. O dióxido de nitrogênio também apresentou associação inversa com o fluxo expiratório forçado entre 25% e 75% da capacidade vital forçada (FEF_25%-75%_) (β -0,30; IC95% -0,58; -0,03).

**Tabela 3:** Coeficiente beta (β) e intervalo de confiança de 95% (IC95%) da análise de regressão entre variabilidade da frequência cardíaca e níveis de poluentes atmosféricos em escolares. Cubatão, 2017 (n=77)

Modelos^b^	Média Diária		Média móvel 2 dias		Média móvel 3 dias	
	β^g^	IC95%^h^	β^g^	IC95%^h^	β^g^	IC95%^h^
SDNN^i^						
MP_10_^c^ (µg/m^3^)	-0,17	-0,56; 0,20	-0,59^a^	-1,10; -0,07	-0,94^a^	-1,72; -0,15
NO_2_^d^ (µg/m^3^)	0,02	-0,12; 0,16	0,07	-0,14; 0,30	-0,01	-0,42; 0,39
O_3_^e^ (µg/m^3^)	0,25^a^	0,02; 0,47	0,30	-0,05; 0,65	0,32	-0,07; 0,72
SO_2_^f^ (µg/m^3^)	0,10	-0,78; 0,99	-0,34	-1,25; 0,56	-0,78	-2,06; 0,49
rMSSD^j^						
MP_10_^c^ (µg/m^3^)	-0,003	-0,31; 0,30	-0,24	-0,56; 0,06	-0,35	-0,75; 0,05
NO_2_^d^ (µg/m^3^)	0,03	-0,10; 0,16	0,04	-0,17; 0,25	-0,03	-0,29; 0,23
O_3_^e^ (µg/m^3^)	0,10	-0,08; 0,29	0,10	-0,14; 0,35	0,06	-0,19; 0,31
SO_2_^f^ (µg/m^3^)	0,003	-0,69; 0,70	-0,36	-1,07; 0,34	-0,82	-1,89; 0,23
LFnu^k^						
MP_10_^c^ (µg/m^3^)	0,07	-0,29; 0,43	-0,22	-0,63; 0,18	-0,30	-0,93; 0,32
NO_2_^d^ (µg/m^3^)	-0,18	-0,38; 0,01	-0,12	-0,39; 0,14	-0,13	-0,44; 0,17
O_3_^e^ (µg/m^3^)	0,14	-0,10; 0,39	0,27	-0,03; 0,59	0,32^a^	0,02; 0,63
SO_2_^f^ (µg/m^3^)	0,68^a^	0,10; 1,27	0,67^a^	0,07; 1,26	1,29^a^	0,38; 2,21
HFnu^l^						
MP_10_^c^ (µg/m^3^)	-0,07	-0,43; 0,29	0,22	-0,18; 0,63	0,30	-0,32; 0,92
NO_2_^d^ (µg/m^3^)	0,18	-0,01; 0,38	0,12	-0,14; 0,40	0,13	-0,17; 0,44
O_3_^e^ (µg/m^3^)	-0,14	-0,39; 0,10	-0,27	-0,59; 0,03	-0,3^2^a	-0,62; -0,02
SO_2_^f^ (µg/m^3^)	-0,68^a^	-1,27; -0,11	-0,67^a^	-1,26; -0,07	-1,29^a^	-2,21; -0,38
LF/HF^m^						
MP_10_^c^ (µg/m^3^)	0,005	-0,02; 0,03	-0,02	-0,05; 0,01	-0,02	-0,07; 0,03
NO_2_^d^ (µg/m^3^)	-0,009	-0,02; 0,003	0,002	-0,02; 0,02	-0,003	-0,03; 0,02
O_3_^e^ (µg/m^3^)	0,008	-0,02; 0,03	0,03	-0,005; 0,06	0,03^a^	0,004; 0,06
SO_2_^f^ (µg/m^3^)	0,06^a^	0,01; 0,12	0,08^a^	0,02; 0,14	0,15 ^a^	0,06; 0,25

a p-valor<0,05; ^b^Modelo ajustado por temperatura média, idade e sexo; ^c^MP_10_: material particulado ≤10 μm em microgramas por metro cúbico; ^d^NO_2_: dióxido de nitrogênio em microgramas por metro cúbico, ^e^O_3_: ozônio em microgramas por metro cúbico; ^f^SO_2_: dióxido de enxofre em microgramas por metro cúbico, ^g^β-coeficiente de regressão estimado pelo modelo; ^h^IC95%: intervalo de confiança de 95% para o coeficiente β; ^i^SDNN: desvio-padrão de todos os intervalos RR normais em milissegundos; ^j^rMSSD: raiz quadrada da média do quadrado das diferenças entre intervalos em milissegundos; ^k^LFnu: baixa frequência em unidades normalizada; ^l^HFnu: alta frequência em unidades normalizada; ^m^Relação LF/HF.

O ozônio mostrou associação positiva significativa com o FEF_25%-75%_ na média móvel de dois dias (β 0,57; IC95% 0,06; 1,08), o que sugeriu possível efeito compensatório sobre a função das pequenas vias aéreas. As variáveis material particulado e dióxido de enxofre não apresentaram associações estatisticamente significativas com os parâmetros espirométricos avaliados nos diferentes modelos de exposição, com coeficientes variando de **β**= -0,28 a 0,05 para material particulado e de **β**= -0,14 a 0,87 para dióxido de enxofre (Tabela 4).

**Tabela 4:** Coeficiente beta (β) e intervalo de confiança de 95% (IC95%) da análise de regressão entre índices espirométricos e níveis de poluentes atmosféricos em escolares. Cubatão, 2017 (n=77)

Modelos^b^	Média diária		Média móvel 2 dias		Média móvel 3 dias	
	β^g^	IC95%^h^	β^g^	IC95%^h^	β^g^	IC95%^h^
CVF^b^ porcentagem do previst^o^i						
MP_10_^c^ (µg/m^3^)	-0,28	-0,82; 0,24	-0,17	-0,57; 0,22	-0,15	-0,66; 0,35
NO_2_^d^ (µg/m^3^)	-0,17	-0,37; 0,02	-0,10	-0,33; 0,12	0,08	-0,23; 0,39
O_3_^e^ (µg/m^3^)	0,06	-0,28; 0,41	0,22	-0,12; 0,58	0,17	-0,15; 0,49
SO_2_^f^ (µg/m^3^)	0,27	-0,25; 0,80	0,58	-0,65; 1,83	0,87	-0,73; 2,48
VEF_1_^b^ porcentagem do previs^t^oj						
MP_10_^c^ (µg/m^3^)	-0,23	-0,75; 0,28	-0,08	-0,49; 0,34	-0,13	-0,75; 0,47
NO_2_^d^ (µg/m^3^)	-0,23 ^a^	-0,43; -0,03	-0,08	-0,34; 0,18	-0,06	-0,26; 0,40
O_3_^e^ (µg/m^3^)	0,14	-0,20; 0,49	0,36	-0,02; 0,74	0,27	-0,09; 0,64
SO_2_^f^ (µg/m^3^)	0,29	-0,20; 0,79	0,80	-0,36; 1,97	0,82	-0,77; 2,42
VEF_1_/CVF^b^ porcentagem do previst^o^k						
MP_10_^c^ (µg/m^3^)	0,07	-0,12; 0,26	0,15	-0,03; 0,33	0,02	-0,37; 0,41
NO_2_^d^ (µg/m^3^)	-0,09	-0,20; 0,02	0,009	-0,12; 0,14	-0,02	-0,18; 0,14
O_3_^e^ (µg/m^3^)	0,09	-0,07; 0,27	0,17	-0,03; 0,37	0,13	-0,08; 0,34
SO_2_^f^ (µg/m^3^)	0,009	-0,18; 0,20	0,27	-0,01; 0,57	-0,14	-0,72; 0,43
FEF_25%-75%_^b^ porcentagem do previst^o^l						
MP_10_^c^ (µg/m^3^)	-0,17	-0,78; 0,43	0,05	-0,58; 0,69	0,02	-0,72; 0,96
NO_2_^d^ (µg/m^3^)	-0,30 ^a^	-0,58; -0,03	-0,007	-0,38; 0,37	0,15	-0,30; 0,62
O_3_^e^ (µg/m^3^)	0,32	-0,08; 0,73	0,57 ^a^	0,06; 1,08	0,40	-0,12; 0,93
SO_2_^f^ (µg/m^3^)	0,07	-0,55; 0,70	0,86	-0,29; 2,02	0,70	-1,11; 2,52

a p-valor<0,05; ^b^Modelo ajustado por temperatura, idade, sexo e altura; ^c^MP_10_: material particulado ≤10 μm em microgramas por metro cúbico; ^d^NO_2_: dióxido de nitrogênio em microgramas por metro cúbico; ^e^O_3_: ozônio em microgramas por metro cúbico; ^f^SO_2_: dióxido de enxofre em microgramas por metro cúbico; ^g^β:coeficiente de regressão estimado pelo modelo; ^h^IC95%: intervalo de confiança de 95% para o coeficiente β; ^i^CVF: capacidade vital forçada; ^j^VEF_1_: volume expiratório forçado no primeiro segundo; ^k^VEF_1_/CVF: índice de Tiffeneau; ^l^FEF_25%-75%_: fluxo expiratório forçado entre 25% e 75% da CVF.

## Discussão

Neste estudo, foi avaliada a associação entre os níveis dos poluentes atmosféricos com a variabilidade da frequência cardíaca e a função pulmonar dos escolares expostos à poluição do ar. Esses resultados demostraram associações estatisticamente significativas entre concentrações crescentes de poluentes e diminuição da variabilidade da frequência cardíaca e dos parâmetros de função pulmonar.

Na variabilidade, foram observados decréscimo nos índices desvio-padrão médio dos intervalos RR (SDNN) com poluentes material particulado ≤10 μm e ozônio, baixa frequência (LF) com dióxido de enxofre, alta frequência (HF) com dióxido de enxofre e, por último, associação positiva na razão entre os componentes de baixa frequência e alta frequência da variabilidade da frequência cardíaca (LF/HF) com ozônio e dióxido de enxofre. Esses achados sugerem que a exposição a poluentes está associada a alterações do ritmo ​​cardíaco em crianças, proporcionada pela disfunção do sistema nervoso autônomo.

Em crianças de 9 a 12 anos, a exposição de curto prazo ao material particulado associou-se à redução da variabilidade da frequência cardíaca. Essa associação foi mais acentuada em crianças com menor conteúdo de DNA mitocondrial [20].

As reduções do índice alta frequência (HF) é associada à retirada do sistema nervoso parassimpático, enquanto os índices desvio-padrão médios dos intervalos RR (SDNN) e baixa frequência (LF) são associados ao comportamento sistema nervoso simpático e parassimpático [21]. Porém, não se pode distinguir quando as alterações da variabilidade da frequência cardíaca são devidas ao aumento sistema nervoso simpático ou à retirada do sistema nervoso parassimpático [17].

A compreensão do comportamento do sistema nervoso autônomo relacionada à exposição à poluição do ar ainda apresenta divergência quanto à diminuição ou ao aumento do sistema nervoso simpático e sistema nervoso parassimpático [22-23]. Esses achados discordantes provavelmente resultam das diferenças nas características dos estudos quanto a exposição ambiental, métodos de estudos, populações abordadas e poluentes analisados [24].

Apesar das divergências sobre o comportamento do sistema nervoso simpático e do sistema nervoso parassimpático, a disfunção do sistema nervoso autônomo é descrito como uma via importante nos mecanismos fisiopatológicos. Estes explicam os efeitos deletérios na saúde causados pela exposição aguda ou crônica a poluentes gasosos e particulados [25], associando-os à alteração no tônus autonômico devido à ativação dos reflexos neurais pulmonares [26].

Considera-se que a maior parte desses achados está relacionada aos estudos em adultos, idosos ou em uma população de risco aumentado, como cardiopatas e pessoas com doenças crônicas [27]. Estudos relacionando variabilidade da frequência cardíaca em crianças são extremamente escassos na literatura [20], o que ressalta a importância destes achados.

Estes resultados para função pulmonar demonstraram associação significativa entre dióxido de nitrogênio e diminuição do volume forçado no primeiro segundo (VEF_1_) e do fluxo expiratório forçado entre 25% e 75% da capacidade vital forçada (FEF_25%-75%_), o que sugere efeito deletério na função pulmonar associado à exposição à poluição do ar.

Resultados semelhantes foram descritos em estudos anteriores [3,28-30] e de outras localidades [10,28-30], além reforçar os resultados descritos na literatura. A maioria dos estudos analisados associou os efeitos da poluição do ar a efeitos deletérios na saúde respiratória das crianças e descreveu partículas e dióxido de nitrogênio como os poluentes com maior associação. Para dióxido de enxofre, não foram verificadas consistências com efeitos à saúde. O ozônio relacionado com a temperatura aparentava um suposto fator protetor para a saúde respiratória das crianças [30]. Esse suposto efeito foi observado nestes resultados para FEF_25%-75%_ com a média móvel de dois dias do ozônio_._

Os efeitos na saúde relacionados à exposição à poluição do ar têm sido observados mesmo quando os poluentes permanecem abaixo dos níveis determinado pela legislação [29]. A diminuição da função pulmonar foi verificada em crianças residentes na cidade do Rio de Janeiro, mesmo dentro de valores considerados aceitáveis pela legislação para os poluentes material particulado ≤10 μm e o dióxido de nitrogênio. Isso indica que os efeitos agudos à poluição do ar na saúde das crianças pode estar presente mesmo abaixo do padrão de qualidade do ar [29]. 

As crianças e os adolescente são considerados mais vulneráveis aos efeitos deletérios causados pela exposição à poluição do ar [1,7]. Fatores de imaturidade do pulmão e do sistema imunológico, junto às maiores taxas de ventilação e atividade ao ar livre de crianças, tornam esse grupo mais suscetível aos efeitos da poluição do ar ambiente [3there are still open questions in the field of health effects of air pollution. One issue is the lack of knowledge regarding health effects of UFP. Furthermore, studies need to disentangle the impact of single pollutants in the complex pollutant mixture that ambient air pollution consists of. The scientific methods also need further development, especially regarding the estimation of individual exposure to ambient air pollution. With the aim of advancing beyond the current knowledge in the field of air pollution health effects, Mauderly et al. (2010]. Em geral, os efeitos adversos da função pulmonar nas crianças são atribuídos à formação reduzida dos alvéolos e à inflamação que promove o remodelamento das vias aéreas causado pela exposição à poluição do ar [28]. 

A função pulmonar na infância é considerada forte preditor da função pulmonar na idade adulta, pois o desenvolvimento da função pulmonar é observado até o final da adolescência no sexo feminino, enquanto, no sexo masculino, pode chegar aproximadamente até os 20 anos, porém em uma proporção mais reduzida do que na infância [3]. 

Nos Estados Unidos, em 2015, foi verificada, na faixa etária 11-15 anos, melhora significante da função pulmonar decorrente da diminuição de poluentes na atmosfera. Sugeriu-se que a melhora no crescimento da função pulmonar observada em crianças menos expostas aos poluentes pode persistir na idade adulta, diminuindo risco de problemas respiratórios [3]. 

Apesar da importância dos resultados encontrados, algumas limitações devem ser abordadas como a não verificação de alguns fatores de confusão como fumo passivo, nível de atividade física dos escolares estudados e tempo de permanência em ambientes externos para os escolares. Outra limitação foi a não verificação dos dias das semanas em que as coletas foram realizadas - isso pode interferir na concentração e na interação dos poluentes com variabilidade da frequência cardíaca e função pulmonar. 

Torna-se necessário mais estudo para compreender a relação dos efeitos à exposição à poluição do ar com variabilidade da frequência cardíaca e função pulmonar em escolares.

## Data Availability

O banco de dados final e consolidado utilizado nesta pesquisa estará disponível mediante solicitação por *e-mail* ao autor correspondente, sujeita à aprovação.
